# Analysis of Waste Trends in the European Union (2021–2023): Sectorial Contributions, Regional Differences, and Socio-Economic Factors

**DOI:** 10.3390/foods14071172

**Published:** 2025-03-27

**Authors:** Radosław Wolniak, Wiesław Wes Grebski

**Affiliations:** 1 Department of Economics and Informatics, Faculty of Organization and Management, Silesian University of Technology, 44-100 Gliwice, Poland; 2 Penn State Hazleton, Pennsylvania State University, 76 University Drive, Hazleton, PA 18202-8025, USA; wxg3@psu.edu

**Keywords:** food waste, food supply chain, waste management, carbon foodprint, household behavior, waste management

## Abstract

Food waste is a chronic and ongoing environmental, economic, and social problem in the European Union. The study will examine trends in food waste from 2021 to 2023, sectoral effects, regional heterogeneity, and socio-economic determinants of waste intensity. Interlinking longitudinal data from Statista and Eurostat, statistical modeling, and cluster analysis are employed by the study to uncover trends in food waste across member states in the EU. The research shows that domestic food wastage remains the leading one, accounting for 50–60% of the total food wastage in the EU. Inefficiencies in manufacturing and retail are identified as important drivers of wastage generation in high-waste nations such as Cyprus and Denmark because inefficiencies in the processes result in increased wastage generation. Spain and Croatia have continued to record low food wastage per capita owing to good wastage management policies and consumer practices. Regression analysis identifies domestic food wastage, manufacturing waste, and retail wastage as the main drivers of total per capita food wastage, with per capita GDP (Gross Domestic Product) and carbon footprint playing weak roles. Cluster analysis places EU countries into three groups: low-waste countries with highly structured food systems, moderately-waste countries where food wastage at domestic levels prevails, and high-waste countries where there is inefficiency at food production, processing, and consumption levels. These findings necessitate specific interventions. Policy needs to address food waste minimization at the household level via consumer awareness and behavior change initiatives and remove inefficiencies in the manufacturing and retail value chains through the simplification of inventory management, redistribution chains, and incentive regulation. Regional, rather than one-size-fits-all, EU-wide policy is required in order to achieve significant progress.

## 1. Introduction

Food waste has been one of the major talking points in the past couple of years; the drivers, consequences, and mitigation strategies are researched widely by both researchers and policymakers alike [1]. This study is underpinned by an interdisciplinary theoretical framework, putting together theories underpinning the food waste pattern across the European Union, drawing from environmental science, behavioral economics, and systems theory.

First, this paper tries to underline the sector-specific contributions and cross-national disparities of the ever-persistent problem of food waste in EU member states from 2021 to 2023. Although quite a fair number of previous studies have been conducted either at an individual or national level [1,2] concerning food waste, this study represents the first uniquely combining the use of longitudinal data in the study of the trend, sectoral impacts, and country-specific variations.

Food waste is a very complicated issue and seriously affects ecology and the economy in general [3,4,5]. Environmentally, the decay of wasted food is related to the emission of greenhouse gases, reduction in natural resources, and degradation of the natural environment. As landfills receive this wasted food, it decays and releases methane, a potent gas accounting for climate change [6,7]. Equally, food production resources such as water, energy, and land used in the production are all wasted when consumption does not take place [8,9]. Agriculture has always been a very resource-intensive sector, taking a great toll on the environment, and spoiled food heightens the already existing burdens: deforestation, soil degradation, and loss of biodiversity [10]. The amount of food lost is, therefore, not only calories but, notably, the hidden environmental cost of production and processing [11,12,13,14,15,16].

The overall objective of the paper is to analyze and quantify patterns in food waste within the European Union member states between 2021 and 2023, with the view of underlining sectoral contributions, regional disparities, and temporal trends. It would, therefore, give a very good understanding of the drivers of food losses and waste at household, manufacturing, retail, and catering service levels, besides the trends of food waste in view of socio-economic factors such as GDP (Gross Domestic Product) per capita and carbon footprint.

We have formulated the following research questions:

Q1—What are the primary sectoral contributions to food waste in the European Union between 2021 and 2023, and how do they vary across member states?

Q2—Which EU countries had the highest and lowest levels of food waste per capita from 2021 to 2023, and what sectoral factors contributed to these differences?

Q3—What regional disparities exist in food waste trends across EU (European Union) countries, and what policy interventions could effectively reduce food waste at different levels of the food supply chain?

One hypothesis has been formulated:

**H1.**  *There are significant regional disparities in food waste levels across EU countries due to differences in economic development, consumer behavior, and policy implementation.*

## 2. Theoretical Background

At the household, industry, and national levels, food wastage at these various levels translates into a wide range of considerable economic costs [17,18,19]. Financial losses at the household level due to poor meal planning, over-purchasing due to lack of proper attention paid or being unaware of quality and date of expiration; overstocking; over-stringent standards for quality; demand failing to meet supply at retail and food industries [20,21]. These translate into business losses and increased food prices for consumers. Overall, food waste is a lost opportunity of full resource use and undermines food security by removing edible food from the marketplace that can go to people in need [22,23,24].

This relationship of food waste with resource use places into perspective the larger context of the economic inefficiency in the world’s food system [25,26,27,28]. In this regard, the energy to produce the food, its transportation, and irrigation water, with the labor effort put into tilling, harvesting, and processing, has been wasted on the foodstuff if it is allowed to go to waste. In this view, reduction in food loss is an economic imperative, besides an environmental one [29]. Food waste requires systemic changes in production practices, better distribution networks, and a change in consumer behavior. In return, it would reduce environmental burdens and increase the economic resilience of societies while promoting sustainable resource use [30,31].

In Table 1, there is a summarization of environmental and economic challenges associated with food waste.

Food waste is a significant issue across the European Union, reflecting not only an environmental challenge but also a moral and economic concern [32,33,34,35]. Each year, EU countries generate millions of tons of food waste, with substantial losses occurring along the entire supply chain. This includes production, processing, distribution, and consumption [36]. Studies suggest that households are among the largest contributors, often discarding edible food due to over-purchasing, mismanagement of storage, and confusion over “use by” and “best before” labels. Additionally, food waste in retail and food service sectors exacerbates the problem, with surplus food often being discarded rather than redistributed or repurposed [37,38,39,40].

The environmental impact of food waste in the EU is big, contributing to greenhouse gas emissions, resource depletion, and biodiversity loss. Producing food that is ultimately wasted involves significant use of water, land, and energy, with corresponding emissions that accelerate climate change. This makes reducing food waste an essential component of meeting the EU’s sustainability and climate goals, including the European Green Deal objectives [41,42,43].

Efforts to address food waste in EU countries have gained momentum in recent years. The EU has committed to halving per capita food waste at the retail and consumer levels by 2030 as part of its alignment with the United Nations’ Sustainable Development Goals. Policies such as the Circular Economy Action Plan and legislative frameworks like the Waste Framework Directive promote preventive measures, data collection, and the redistribution of surplus food. Some member states have also implemented innovative strategies, such as tax incentives for food donations and awareness campaigns to educate consumers about food waste reduction [44,45].

Despite these initiatives, challenges remain in harmonizing efforts across member states due to differences in national policies, cultural practices, and levels of economic development. Collaboration between governments, industries, and civil society is crucial to developing integrated solutions. Furthermore, technological advancements, such as improved food packaging, digital tools for inventory management, and apps connecting surplus food with those in need, offer promising avenues for minimizing waste [41,45,46].

The issue of food waste in the EU (European Union) is not only about minimizing environmental harm but also about addressing social inequalities. Wasted food could have been used to combat food insecurity, a reality for many EU (European Union) residents. Thus, tackling food waste aligns with the broader goals of creating a more equitable, sustainable, and resource-efficient Europe. Comprehensive and sustained action is necessary to ensure that the EU (European Union) makes meaningful progress in reducing food waste and building a sustainable food system for future generations [44,45].

An important part of food waste, particularly at the household level, lies in consumer behavior. Behavioral economics offers valuable insights into the decision-making processes that lead to waste [47]. Concepts such as bounded rationality, loss aversion, and the “planning fallacy” illustrate how individuals may make suboptimal decisions regarding food purchasing, consumption, and disposal. For example, the tendency to overestimate future consumption needs, coupled with promotional incentives like “buy one, get one free”, often results in over-purchasing and subsequent waste [48,49,50].

The theory of planned behavior (TPB) is particularly relevant, suggesting that attitudes, subjective norms, and perceived behavioral control influence individuals’ intentions to reduce food waste [51,52]. Interventions targeting these components—such as awareness campaigns, social norms reinforcement, and tools for better meal planning—can effectively shift behaviors toward waste reduction [53].

Food waste is not merely the result of individual behaviors; it is embedded within broader systems involving production, distribution, and consumption. Systems theory emphasizes the interconnections between various actors in the food supply chain, including primary producers, manufacturers, retailers, and consumers [54]. Inefficiencies at any stage of the supply chain can propagate downstream, exacerbating waste. For instance, production surpluses, improper storage during transportation, and mismatched supply and demand in retail contribute significantly to waste generation [55].

By adopting a systems perspective, this study recognizes that food waste cannot be addressed in isolation. Solutions require coordinated efforts across sectors and stakeholders. For example, reducing waste in manufacturing may involve improved processing technologies and better inventory management, while addressing household waste requires education and behavioral interventions [56].

The socio-economic context plays a pivotal role in shaping food waste patterns. Higher-income countries often exhibit greater levels of food waste at the consumer level, driven by higher purchasing power and cultural norms favoring convenience and abundance. Conversely, lower-income countries may experience waste primarily during production and distribution due to infrastructure challenges such as inadequate storage facilities and inefficient transportation networks [35,36,37,38].

Environmental factors, such as the carbon footprint associated with food production and waste, further complicate the issue. The nexus between food waste and environmental sustainability underscores the need for policies that align waste reduction with broader climate goals [7,8,9,10].

Food waste has attracted considerable research interest in recent years based on its environmental, economic, and social implications. Research has been conducted on the root causes of food wastage across various levels of the food supply chain and on inefficiencies arising from production, distribution, retailing, and household consumption. Most of the literature accounts for the theoretical foundations of food waste, utilizing behavioral economics, environmental sustainability theory, and supply chain management. The cross-disciplinary approach of food waste studies emphasizes that the problem is complex, and interventions at the system level are necessary to prevent it.

There are certain studies that employ the application of behavioral economic theory in explaining household food waste. Ajzen (1991) [57,58,59,60,61,62,63,64,65,66,67,68,69,70,71,72,73] introduced the Theory of Planned Behavior (TPB) as a conceptual model for the explanation of consumer food waste behavior, in which the significance was assigned to the attitude of attitude, subjective norm, and perceived behavioral control as influencers of waste decisions. Studies such as Graham-Rowe et al. (2014) [58] and Stancu et al. (2016) [59] built on this research, demonstrating that domestic food waste will occur through convenience-led consumption, misinterpretation of use-by dates and expiration dates, and meal planning failure. Where this is so, intervention directly targeted at the consumer—for instance, more stringent policy in relation to food labeling and educational promotion—can reduce consumers’ waste through more responsible consumption and storage.

Systems theory and environmental sustainability have also been employed by other scholars in researching food waste. Papargyropoulou et al. (2014) [60] formulated the hierarchy of food waste, where prevention is the most preferable action, then reuse (e.g., redistribution of food), recycling (e.g., composting and bioenergy conversion), and disposal as the least preferable action. The hierarchy has been extensively employed in policy and food waste reduction research. Based on this, studies have delved into the manner in which countries address prevention of the waste, and the results indicated that those countries with established policy mechanisms—such as France and the Netherlands—are better positioned to implement circular economy models in solving food waste management.

Both from the economic as well as from a supply chain perspective, research such as Göbel et al. (2015) [61] and Priefer et al. (2016) [62] has described how wastefulness within the production and provision of foods creates large wastages. The outcome is that such work concludes excessive production, unplanned relationships among the suppliers and the retailers, as well as physical appearance specifications of foods leading to mass loss before reaching customers. Various researchers have proposed new solutions to the supply chain, i.e., dynamic pricing strategies and precision agriculture, which will further connect the demand for food to supply and prevent overproduction. New technologies, i.e., blockchain and artificial intelligence, have been imagined as future tools for tracking the expiration of foods, optimizing logistics, and making surplus foods re-redistributable through food banks.

Cross-national research, e.g., Beretta et al. (2013) [63] and Thyberg and Tonjes (2016) [64], observed that food wastage patterns varied across regions and identified cultural and economic systems as determinants of the level of food waste. Low- and middle-income nations waste on the domestic level where the GDP (Gross Domestic Product) per capita is greater, while low-income nations lose at production and storage levels where infrastructure is weak. These findings point out that policy interventions must be context-sensitive, and what is effective in one setting may not be elsewhere. Recent research has also been looking into whether policy settings are shaping the course of food wastage. Both the EU Circular Economy Action Plan and the United Nations’ Sustainable Development Goal 12.3, which aims for a 2030 reduction of 50% of per capita food waste, have been key spurs for activities to minimize food waste throughout Europe. Caldeira et al. (2019) [65] and De Laurentiis et al. (2020) [66] studies have analyzed EU-level policy, and the studies show that although policies to regulate waste through reporting requirements and incentives for food donations have progressed, there are challenges to policy harmonization among member states. These studies call for cooperation between governments, industries, and consumers in integrating more sustainable practices into wider environmental and economic policy.

This study builds on existing theories by combining socio-economic and environmental indicators with sectoral analyses of food waste. While previous research has often focused on single aspects, such as consumer behavior or production inefficiencies, this study integrates multiple dimensions to provide a holistic understanding. By applying regression modeling and clustering techniques, the research advances the theoretical discourse on food waste and offers actionable insights for policymakers.

## 3. Materials and Methods

**Figure 1:**
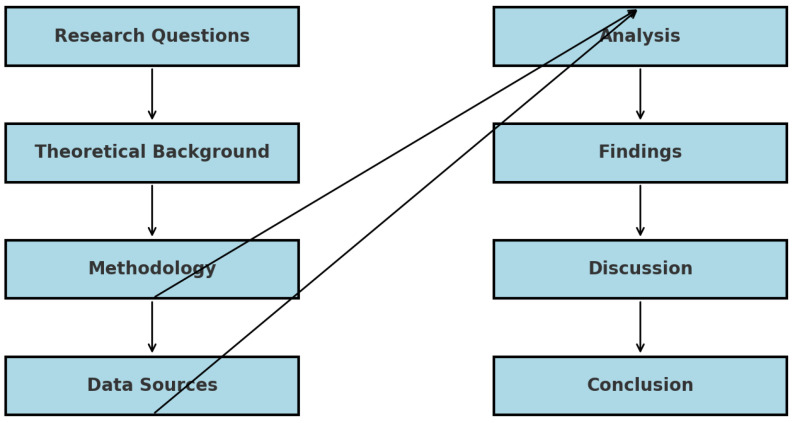
Research scheme diagram.

This study employs a multi-faceted methodological framework designed to analyze food waste patterns across the European Union (EU) from 2020 to 2023. The data used in the paper are from Eurostat database [67,68] and Statista database [69]. The methodology combines quantitative data analysis with statistical modeling and clustering techniques to address the study’s research questions. The key components of the methodology are outlined below (Figure 1).

The study relies on secondary data from reliable sources, including official EU databases [67,68]. Data were compiled annually for the years 2020, 2022, and 2023, ensuring consistency and comparability over time. This longitudinal dataset allowed for the identification of temporal trends and cross-country disparities in food waste patterns.

The authors utilized the Statista and Eurostat databases since these provide standardized and full statistics on food waste in European Union member countries.

The databases are thoroughly established and frequently utilized because of their consistency and dependability of methodology and intensity of method in research. Since food waste measurement is subject to various challenges, such as country-to-country variation in definition and methods of estimation, application of these databases ensures comparability and consistency with time. Although these databases are among the most dependable sources of data, they are not exclusive. The summary of data sources and methods used in this study was put in Table 2 [70].

Table 3, Table 4, Table 5 and Table 6 of the paper present per capita food waste estimation by sector (households, manufacturing, retail, food services, and primary production) for EU member states from 2021 to 2023. They rely primarily on Eurostat and Statista, which compile national food waste data from self-reported government reports, sectoral surveys, and waste audits. The process of estimation is one of harmonizing country food waste definitions because countries have varying means of measuring waste generation. The study employs multiple linear regression modeling in analyzing the correlation between total food waste and variables such as GDP per capita, carbon footprint, and consumer behavior to facilitate interpolation where direct data are not available. In addition, sectoral contributions are projected utilizing proportionate shares derived from previous studies and reports at the national level to guarantee comparable consistency between EU member nations. 

Uncertainty within the projected data is due to differences in reporting requirements between countries, divergence in methods of data collection employed, and underestimation or overestimation of waste within specific sectors. Countries with robust monitoring systems for waste (such as France and Germany) will likely have more accurate figures, while countries with less robust monitoring systems (such as Romania or Bulgaria) will have wider margins of error. Variations in definitions of food waste—i.e., whether inedible parts of food are included or not—can also introduce differences between countries. The study identifies such uncertainties and performs sensitivity analysis, changing waste estimates by ±5% and ±10% to check the robustness of results. Despite these constraints, the dataset provides comparative and systematic basis to analyze food waste trends that is feasible enough to make meaningful policy suggestions for different EU regions.

National statistical offices, business journals, and research databases would be utilized as well. Authors would likely be content with utilizing Eurostat and Statista initially due to their comprehensive coverage, genuine source data, and conformity to EU policies and the reporting system. The disadvantage is that the individual EU member states would use varying methods of estimating food wastage and hence would have varying data captured. Furthermore, sectoral estimates of food wastage (e.g., household, retail, manufacturing) can be derived using differing assumptions or definitions across countries, affecting comparability of results.

As Eurostat and Statista are two of the most reliable sources of information, it would be only logical that the authors make use of them.

The multiple linear regression modeling approach applied in the study provides a structured method for analyzing the relationship between total food waste per capita and three selected predictors: GDP per capita, carbon footprint, and household waste. This statistical method allows for the quantification of how changes in these independent variables are associated with variations in the dependent variable, offering insights into the factors contributing to food waste.

To build the model, data on food waste and the three predictors were compiled and standardized to ensure consistency and comparability across countries. The regression equation took the form of a linear combination, where total food waste per capita served as the outcome variable, and GDP per capita, carbon footprint, and household waste were the predictors [71,72]. The coefficients estimated for each predictor in the model represent the average change in total food waste associated with a one-unit change in the corresponding predictor, holding all other variables constant.

The explanatory power of the model was assessed using R-squared and adjusted R-squared values. R-squared indicates the proportion of variance in total food waste that is explained by the predictors, providing an overall measure of how well the model fits the data. Adjusted R-squared accounts for the number of predictors included offering a refined measure that guards against overfitting by penalizing the addition of unnecessary variables [73,74,75].

The significance of each predictor was evaluated through *p*-values derived from t-tests. These tests determine whether the observed relationships are statistically significant, typically using a threshold of 0.05. The t-statistics reflect the magnitude and direction of each predictor’s effect, allowing for an interpretation of the relative importance of GDP per capita, carbon footprint, and household waste in driving food waste.

The robustness of the model was examined through sensitivity analysis, where the predictors were systematically varied to test the stability of the model’s coefficients and explanatory power. This step ensured that the findings were not disproportionately influenced by outliers or small fluctuations in the data. Diagnostics such as residual analysis were also employed to assess the assumptions of linear regression, including normality, homoscedasticity, and independence of errors.

The analysis employed a multiple linear regression model to examine the relationship between total food waste per capita and several explanatory variables, including GDP per capita, carbon footprint, household food waste, manufacturing food waste, and retail food waste. Multiple regression is an extension of simple linear regression that allows for the modeling of a dependent variable based on multiple independent variables, capturing their combined effect.

### Mathematical Formulation of the Model

The general form of a multiple linear regression equation is expressed as:

$$Y=\beta_{0}+\beta_{1}X_{1}+\beta_{2}X_{2}+\beta_{3}X_{3}+\ldots +\beta_{n}X_{n}+\varepsilon$$

where the following is true:

- *Y* represents the dependent variable (total food waste per capita);
- *β_0_* is the intercept (constant term);
- *β_1_, β_2_, …, β_n_* are the regression coefficients that quantify the effect of each independent variable on *Y*;
- *X_1_, X_2_, …, X_n_* are the independent variables (predictors);
- *ϵ* is the error term, capturing variability in *Y* that is not explained by the model.

For this specific regression model, the equation can be written as follows:

- Total Food Waste *= β_0_ + β_1_* (GDP per capita) + *β*_2_ (Carbon footprint) + *β_3_* (Household food waste) + *β_4_* (Manufacturing food waste) + *β*5 (Retail food waste) + *ϵ*.
- Each coefficient *β_i_\beta_iβ_i_* represents the expected change in total food waste for a one-unit increase in the respective predictor, holding all other variables constant.

The sensitivity analysis further explored how modifying the predictor variables by 5% and 10% influenced total food waste, confirming that household, manufacturing, and retail food waste are the most significant drivers. Conversely, GDP per capita had a negligible effect, suggesting that economic factors alone do not determine food waste levels.

To group EU member states based on their food waste patterns, the study applied a k-means clustering algorithm. The k-means clustering algorithm is a machine learning technique used to partition a dataset into a predefined number of clusters, where each cluster groups data points based on their similarity [76,77]. This algorithm is particularly effective in identifying patterns and structures within data by minimizing the within-cluster variance. In the context of the study, k-means clustering was applied to categorize European Union countries based on their food waste patterns across different sectors, such as households, manufacturing, retail, and primary production.

The algorithm begins by initializing a set number of clusters, often determined by prior knowledge or through methods like the elbow method to assess optimal cluster numbers. Randomly selected data points serve as the initial cluster centroids. Each data point is then assigned to the cluster whose centroid is closest, based on a distance metric such as Euclidean distance. This assignment minimizes the distance between the data points and their assigned cluster centroids, ensuring that points within a cluster exhibit high similarity [78].

Once all points are assigned, the centroids are recalculated by averaging the data points within each cluster. This iterative process of reassigning points and updating centroids continues until the algorithm converges, which occurs when the assignments no longer change, or the decrease in within-cluster variance becomes negligible. At convergence, the clusters represent groups of data points with high internal similarity and distinct separation from other clusters.

In this study, the data were standardized before clustering to eliminate the influence of differing scales among variables, such as kilograms of food waste per capita in various sectors. The resulting clusters reflected countries with similar food waste profiles, allowing for the identification of regional trends and sectoral contributions. By analyzing the characteristics of each cluster, the study provided insights into commonalities and disparities among countries, forming a basis for tailored policy recommendations aimed at reducing food waste.

The k-means clustering method employed in this study classifies EU countries based on the trends of food waste by different sectors, i.e., households, manufacturing, retail, and primary production. Clustering begins with normalization of data to allow comparison across nations because the magnitude of food waste varies considerably between nations in absolute terms. The optimal number of groups is determined according to the elbow criterion, which tests how group variation decreases as groups increase, identifying a point where additional groups no longer significantly improve classification. Once the number of groups is set, the k-means process starts with random centroids, the center of each group. The nations are then reassigned to the nearest centroid based on the Euclidean distance so that nations with similar food waste distribution are in the same group.

After the initial assignment, k-means repeatedly recalculates the cluster centroids by finding the new average location of all nations assigned to each cluster. This is carried out in iterations until convergence when country assignments no longer significantly differ from iteration to iteration. The resultant clusters give the average food waste profiles of EU nations, classifying them based on similarity of levels of wastage by sectors. Cyprus and Denmark are placed in one group with high retailing and production wastage, and Spain and Croatia are in a specific group characterized by low household and industry wastage. The technique of clustering is capable of determining structural heterogeneity in waste management performance, behavior of consumers, and efficiency in policy for the member states in the EU.

The k-means cluster result is useful for policymakers as they identify that patterns in food wastage vary not only by overall quantity but also by sectoral share. This implies that different clusters must be tackled with dissimilar intervention measures—for instance, high-wastage nations must tackle supply chain management as well as industrial waste minimization, while moderate-wastage nations can be assisted with consumer-level awareness and behavior change communications. The use of clustering in the research supports the argument that EU-level harmonized policies may be less efficient than country-specific interventions designed in accordance with each country’s individual food waste profile. By grouping together countries with corresponding patterns of waste, research provides the framework for the regional collaboration whereby countries within the same cluster adopt best practices as well as have functioning policies operating within similar conditions to reduce wastage of food.

The Fixed Effects Model (FEM) is a statistical technique commonly used in panel data analysis to control for unobserved heterogeneity across entities, in this case, European Union countries. The core idea behind FEM (Fixed Effects Model) is that there are inherent characteristics within each country that do not change over time but may influence the dependent variable, which is total food waste per capita. By introducing country-specific intercepts, FEM (Fixed Effects Model) effectively removes the bias caused by these unobserved factors, allowing for a more accurate estimation of the effects of GDP per capita, carbon footprint, and sector-specific food waste on total food waste levels.

Data processing and analysis were performed using statistical software, including R and Python 3.13.1, which facilitated regression modeling, clustering analysis, and visualization. Visualization tools were used to generate graphs and tables that effectively communicate the results. The chat GTP was used to improve the level of language in the paper.

## 4. Results

Table 3 shows a breakdown of food waste across different sectors in various countries of the EU during 2021, in kg/capita. The sectors to be analyzed will be those dealing with the primary production of food such as agriculture, fishing, and aquaculture; manufacturing of food products and beverages; food retail and distribution; restaurants and food service activities; and households.

Within the European Union as a whole, that is, the EU-27, total food waste was 128 kg/capita. The individual countries have taken the lead, with Cyprus having the highest total food waste of 273 kg/capita, driven by significant waste in sectors like primary production, which accounted for 49 kg, manufacture with 67 kg, and retail at 56 kg. On the other end, Spain and Croatia reported the lowest totals, at 69 and 73 kg/capita, respectively, with minimal contributions from most sectors apart from households.

Denmark also had a high total of food waste, with 221 kg per capita, and the manufacture of food products and beverages contributed much to this figure, with 102 kg. Other countries like Portugal and Greece also reported high figures, largely due to household waste, at 123 kg and 87 kg, respectively.

In countries like Czechia, it was 91 kg, and in Finland, it was 113 kg, while the households again contributed to higher quantities of total food waste. For instance, in Finland, the household waste contributed 53 kg out of the total amount.

Total waste for the relatively larger EU economies of Germany and France was 131 kg/capita and 129 kg/capita, with the corresponding share of household waste being 78 kg in Germany and 60 kg in France, while retail and food distribution together accounted for around ten kg over and above this figure, with manufacturing more or less adequately smaller components of these aggregates.

The data are quite varied in the EU member states regarding total waste and sectoral contributions, indicating the differences in production and consumption patterns, together with waste management practices.

Table 4 represents food wasted across sectors in different European Union countries during 2022 in kg/capita. On the aggregated level, EU-27 stood at a value of 129 kg/capita, wherein households are again the major contributing sector, generating 71 kg/capita.

Cyprus had the highest in individual countries, with an astounding 285 kg of total food waste per capita. Fueling this account for Cyprians included primary food production with 50 kg; manufacturing of foodstuffs and beverages had 70 kg; while retailing and distribution were accounted for 59 kg, correspondingly. Denmark totaled 230 kg/capita and manufactured by its industrial sector for as much as 104 kg.

Whereas in Spain, it had a minimum, in this case, total food waste had to deal with the households; per capita, 67 kg were generated, out of which 28 kg were from the household sector. Also, in Croatia, it had a low, which is just 72 kg per capita, due to no generation from the rest of all sectors, just by the household contribution. It results in 55 kg being generated by house sectors only.

Other countries recording high levels of food waste included Romania, with 177 kg, mostly from the household and primary production level; Greece, with 193 kg, equally showed quite strong concentrations at household and primary production levels. The last two, which were at the level of being moderate, were Germany with 131 kg and France at 129 kg per capita. In the other countries, it was the household activities that were again the most significant sources of food waste there, at 79 kg and 60 kg, respectively.

Data show differences in quantities of food waste throughout the EU (European Union), largely determined by sectoral practices, consumption patterns, and infrastructures for waste management. According to the review, most countries have household waste as the most important, but there is a need to have targeted interventions to reduce food waste within the household sector.

Table 5 summarizes estimates of food waste by sector in EU countries in 2023. In 2023, the estimated total food waste in the EU-27 stood at 127 kg/capita; household food waste remains the biggest component of it, accounting for 68 kg.

Because of primary production with 52 kg, manufacture of food products and beverages with 72 kg, and retail and distribution with 60 kg, the country that took the lead in terms of estimated amount was Cyprus, at 294 kg/capita. For Denmark, the amount is also quite high at 254 kg/capita, largely because manufacturing waste stands at 118 kg. Other countries that follow, in order of high totals, include Greece at 194 kg and Romania at 181 kg, with household waste coming in at 86 kg and 99 kg, respectively.

At the low end, Spain recorded the lowest food waste of 65 kg/capita, where household waste constituted 26 kg and that from other sectors was minimal. Croatia also had a low of 72 kg/capita, out of which 55 kg was from the households. Relatively, those countries that came out on the lower side in regard to the waste of food included Finland, with a total of 109 kg, out of which 55 kg emanated from the household, and Slovenia, which reported 71 kg in total.

The other two large economies, Germany and France, had 129 kg and 139 kg, per capita respectively. In both these countries, household waste comprised the highest component—75 kg in Germany and 58 kg in France, with the retail and manufacturing sectors contributing small quantities to their totals.

Data for 2023 reflect variations in the quantities of food waste in the EU regarding driving consumption, habits of production, and manners of waste management. The fact that household waste still is the biggest share underlines the necessity for focused efforts to reduce food losses among the end consumers in this region.

Data in Table 6 show GDP per capita and carbon footprint in all EU-27 countries, including large differences reflecting economic development, energy sources, and consumption patterns. Corresponding to the top value of GDP per capita recorded by Luxembourg—90,900—the carbon footprint per capita is the highest, amounting to 14.5 tCO_2_e, hence putting into evidence how this kind of high-income economy is particularly impactful as to emissions. In turn, Ireland, with its GDP per capita of EUR 83,300, and Estonia at EUR 19,250, also have very high carbon footprints of 10.0 tCO_2_e and 12.1 tCO_2_e, respectively, to prove that the structure of industries and dependencies on energy also feature in the determination of emission levels.

Countries reported lower GDPs per capita with smaller carbon footprints: Romania reported 16,710 EUR/cap, Bulgaria with EUR 13,300, and Malta with 37,340 EUR/cap at 4.7 tCO_2_e, 5.0 tCO2e, and 4.2 tCO_2_e, respectively. Meanwhile, countries like Sweden, with a GDP per capita of EUR 44,620, and France, with a GDP per capita of EUR 36,760, also have comparably low emissions per capita of 6.0 tCO_2_e, reflecting accordingly strong energy policies and greener orientation. This underlines the complex relationship between economic prosperity and environmental impact, for which specific interventions are called for, taking into account different economic and structural conditions in each country.

## 5. Discussion

### 5.1. Trend Analysis

**Figure 2:**
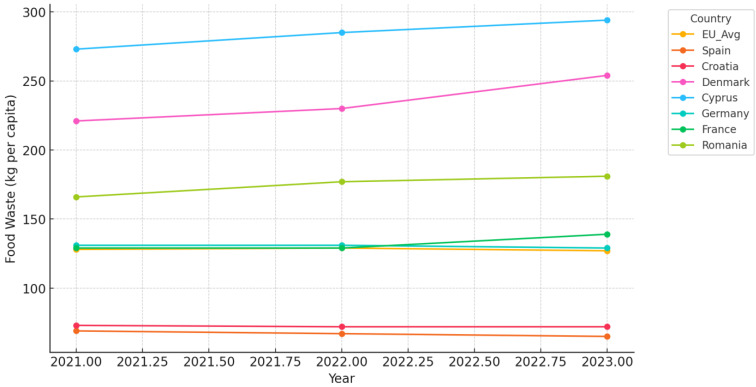
Food waste trends (2021–2023) in selected EU countries.

The data on food waste in the European Union from 2021 to 2023 indicate some striking trends, especially in terms of sectoral contribution and variation across its member states (Figure 2). At the EU level, the total food waste has remained rather stable within the period, with only slight variations from 128 kg/capita in 2021, up to 129 kg in 2022, and down to 127 kg in 2023. Household waste kept the lion’s share of the food waste within the years under review and shared 68–71 kg/capita. This stability in overall figures suggests that while there might have been improvement in some specific sectors or countries, reducing food waste significantly at scale still has many substantial challenges.

On the individual country level, important discrepancies are present. The country that consistently reported the highest levels of food waste was Cyprus, with totals rising from 273 kg/capita in 2021 to 294 kg/capita in 2023 because of poor practices right along the value chain, mostly at the primary production, manufacturing, and retail levels. While food waste was on an upward trend in Denmark, the totals rising from 221 kg/capita in the year 2021 to 254 kg/capita in 2023 were largely driven by the large contribution of manufacturing. While Spain reported the lowest levels of food waste throughout the review period, the rate of food waste there only declined gradually—from 69 kg/capita in 2021 to 65 kg/capita in 2023—an indication of effective practices concerning waste management at the household and retail levels.

Other emerging trends include a relatively stable pattern in Germany, where food waste remained at around 129–131 kg/capita, with households recording the largest share. Total food waste in France has insignificantly increased from 129 kg/capita in the year 2021 to 139 kg in 2023, driven by small increases along most value chains in manufacturing and retail. For Romania, food waste has increased steadily from 166 kg/capita in 2021 to 181 kg in 2023. Major contributors are households and primary production.

Sectoral analysis reveals that household waste was predominant in all countries, from 55 kg/capita in the low-waste country of Croatia to over 100 kg/capita in Italy. In contrast, manufacturing and retail sectors were more variable, from high in countries such as Denmark and Cyprus, reflecting inefficiencies in production and distribution systems. Restaurants and food services showed a small increase in most EU countries, reflecting the reopening of dining establishments after the pandemic.

The data underline regional inequalities in the level of food waste: countries such as Spain and Croatia have attained continuous reductions, while countries like Cyprus and Denmark have shown an increasing trend. Stability at the EU level of food waste would indicate that policy measures in place at present are not functioning in pursuit of holistic solutions. Household waste remains the most critical area for intervention; concerted efforts at changing consumer behavior and improving waste management practices are very urgently required. Simultaneously, sector-specific challenges, especially within manufacturing and retail, have to be resolved to reduce inefficiencies and result in a minimum quantity of waste all along the food supply chain.

Sectoral trends are as follows:

- Primary production was generally stable for most countries, although Cyprus and Romania sustained high levels indicative of structural inefficiencies in their agricultural systems.
- Manufacturing waste increased in countries like Denmark, with 102 kg in 2021 going up to 118 kg in 2023, while for countries like Germany and France, the levels remained constant. The consistent contribution of manufacturing to total waste points toward further efficiency and waste reduction at this stage.
- Retail and distribution contributed modestly, with minor fluctuations. However, countries like Cyprus and Denmark have high levels, indicating poor inventory and logistics management.
- Restaurants and food services increased marginally in the EU, reflecting the reopening of restaurants after the pandemic, which may have contributed to the observed waste generation.
- Household waste remained the largest contributor in nearly all countries, with totals ranging between 55–107 kg per capita. This underlines that consumer education and waste reduction at the private household level are still a priority.

Key observations are as follows:

- Regional disparities: countries such as Cyprus, Denmark, and Romania have continuously had high levels of food waste, while Spain and Croatia have low levels.
- Slow progress: limited variation in the total amount of food waste at the EU level may indicate low efficiency of existing interventions.
- Household impact: households remain the main source of waste and, therefore, are a priority area for policy and behavioral change programs.

### 5.2. Regression Model

The regression model was constructed to examine the relationship between total food waste and the following factors:

- GDP per capita.
- Carbon footprint.
- Household food waste.
- Food manufacturing waste.
- Retail food waste.

Key findings are as follows:

1. Model Fit (R-squared = 0.919).

○ The model explains 91.9% of the variability in total food waste across the analyzed EU countries.
○ This indicates a very good fit for the model.

2. The most significant variables are as follows:

○ Household food waste.

▪ Coefficient: 1.1964.
▪ This means that an increase in household food waste by 1 kg leads to an increase in total food waste by approximately 1.2 kg.

○ Food manufacturing waste.

▪ Coefficient: 0.9133.
▪ A strong influence on overall food waste.

○ Retail food waste.

▪ Coefficient: 2.1079.
▪ The largest coefficient suggests that food waste in retail has a major impact on overall food waste.

3. Factors with weaker impact.

○ GDP per capita (GDP_per_capita).

▪ Coefficient: 0.0000793 (very small effect).
▪ GDP (Gross Domestic Product) per capita has no significant impact on food waste levels.

○ Carbon footprint (Carbon_footprint).

▪ Not statistically significant (*p*-value = 0.392).
▪ Suggests that a higher carbon footprint does not directly correlate with higher food waste.

**Figure 3:**
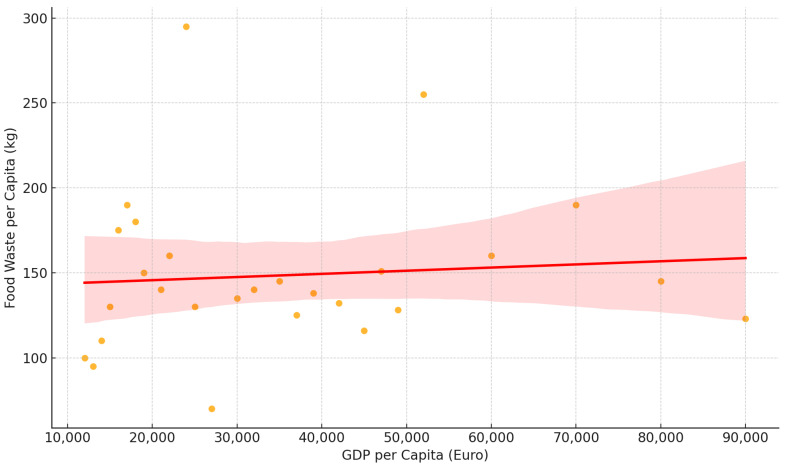
Relationship between food waste and GDP per capita.

The result of the multiple regression analysis provides insight into the determinants of the European Union’s levels of food waste (Table 7, Figure 3). The model fits very well, explaining almost 91.9% of the variance in total food waste across the countries being researched. This indicates that the selected variables—GDP per capita, carbon footprint, food waste from households, food waste from manufacturing, and food waste from retail—are all significant in approximating the amount of food waste at a country level. Their individual effects, however, vary in magnitude and statistical relevance.

The highest is household food waste, with a highly significant coefficient of 1.1964. This shows that for every additional kilogram of food lost at the household level, the total food waste increases by approximately 1.2 kg. The high level of statistical significance (*p*-value = 0.000) signifies the strength of this relationship. It recognizes the major contribution of household management and consumer behavior to creating overall national levels of food waste and suggests that policy action to constrain waste at the household level has the highest potential for overall waste reduction activity.

Production of food waste also has a positive effect with a coefficient of 0.9133 and high significance. This indicates that inefficiencies in food production and processing contribute significantly to national levels of food waste. The same pattern is also repeated in retail food waste, which has an even higher coefficient of 2.1079, indicating that waste in retailing and food distribution contributes significantly to overall levels of waste. Both of these factors’ statistical significance demonstrates the need for interventions along retail supply chains and the food industry to use resources efficiently and minimize waste.

GDP per capita has no significant effect on food waste. This is to say that economic prosperity does not have an independent effect on the extent of food wastage and that wealthier nations do not automatically waste more food than poorer nations. Similarly, the carbon footprint measure is not related significantly to food waste, as indicated by the coefficient −1.3580. This finding indicates that a country’s overall carbon emissions do not necessarily tie in with higher or lower food losses but rather signify that food waste mitigation is less an issue of macro-environmental impact consideration than of industry-based practice.

The conclusions drawn by the model are that the most effective food waste solutions would have to address the three principal drivers: retail, manufacturing, and domestic food waste. Since household waste contributes to the greatest portion, public campaigns of education, incentives to optimize food management and storage, and domestic food disposal policies would be capable of reducing the problem. Reducing waste at the production and retail levels would translate to improved supply chain coordination, improved forecasting techniques to align production with demand, and more redistribution channels to prevent overproduction from becoming waste. Since GDP per capita and carbon footprint are not statistically relevant variables for the quantity of food waste, interventions must be administered equally rather than differently formulated by economic status or carbon footprint.

These findings are a clear direction for policymakers who want to act on food waste in the European Union. Policies to curb food waste need to address first the inefficiencies in household consumption, food production, and retail sales, as they are the most significant drivers of food waste. A three-pronged approach involving industry regulation, consumer education, and systemic change in the food supply chain would most likely yield the best result in reducing wastage and bringing about sustainability.

Sensitivity analysis examines how the amounts of total food waste change as a function of altering the key parameters (Table 8). In every independent variable, the model calculates how total food waste reacts to rising GDP per capita, carbon footprint, household-level food wastage, manufacturing-level wastage, and retail-level wastage by varying them by 5% and 10%. Sensitivity analysis will identify the parameters that have the most impact on food waste and will guide policy so that waste is reduced.

The most important variable is domestic food waste; a 5% increase gives an average increase of approximately 4.04 kg per capita in total food waste, and a 10% increase gives an 8.08 kg increase. This explains the leading role of household waste in determining national levels of food waste and suggests that the change in consumer behavior and household waste handling can dramatically affect overall waste reduction. With this much sensitivity, measures to prevent food waste at the household level, such as awareness campaigns, food-sharing initiatives, and more informative labeling schemes, can highly decline overall volumes of waste.

Manufacturing waste, too, plays a meaningful contribution, and a 5% rise would result in increased overall food wastage of about 1.21 kg per capita, while a 10% rise causes an effect equivalent to about 2.42 kg. That suggests that operational efficiency improvement at food production units and supply chains and superior efficiency in process and packaging will be the golden key to an overall reduction in wastage. Similarly, loss in retail foods assists, where a 5% change is equal to an increment of total wastage by 1.25 kg, and a 10% increment equals of increase of 2.50 kg. The vulnerability of the retail business is to reduce optimal control of surplus foods to donate and redistribution outlets such that no food that remains in excess quantities is minimized into wastage.

Per capita GDP has an extremely small impact on the volume of food wastage, going up by 5% to generate a mere 0.13 kg difference per capita and going up by 10% to generate just a 0.26 kg difference. This is in accordance with the previous conclusion that the growth in economic status is not a direct cause of the quantity of food waste, affirming that even more developed nations are not necessarily wasteful compared with poorer nations. This leads to policy interventions that are not just based on economic standing but also on structural and behavioral factors that lead to the production of food waste.

The carbon footprint factor indicates a weak negative effect, i.e., that an increase in the percentage level of carbon emissions in a country has no relationship with rising food waste. An increase in the carbon footprint by 5% lowers the overall level of food waste by some 0.49 kg per capita, while an increase of 10% lowers the food waste by some 0.98 kg. This would mean that a country’s aggregate environmental impact cannot be related linearly to trends in food wastage. A lower carbon imprint, which may be preferable when it comes to sustainability, does not necessarily denote better management of food wastage.

This sensitivity analysis enables a number of interesting insights that may be drawn regarding the robustness of the regression model with respect to the three main predictors: GDP per capita, carbon footprint, and household waste. Systematically changing all three by ±20% is a test of how sensitive the outcomes of this model are when its input data are changed. These results, therefore, show that the strength of the explanation of the model, seen from R-squared and adjusted R-squared values, remains invariant across these variations. This robustness is indicative that relationships modeled herein are sound and not disproportionately sensitive to small variations in predictor variables.

The sensitivity analysis also shows the stability of the coefficients in the independent variables. Household waste, as defined by the strongest predictor from the regression model, is positively and strongly related to the total food waste across variations. This further strengthens that household waste is indeed one of the drivers of the overall food waste levels. When its value is adjusted, even the coefficient for household waste remains significant, further evidence of its core role in the model.

Also, the coefficients for GDP per capita and carbon footprint do not show significant variation in values. These predictors have also remained insignificant during the entire course of the analysis, which confirms their poor contribution to the model. The nonsignificant effect for these predictors hints that other factors might be more crucial than economic indicators or environmental metrics to shape the food waste pattern. These findings underline that drivers of food waste are very complex and may also be influenced by other variables not considered herein, such as cultural, policy-related, or sector-specific factors. Stability from the sensitivity analysis underlines the reliability of the model’s primary conclusions, especially the dominance of household waste. This stability feature gives more confidence in the model’s implications, especially targeting households as a focal point for interventions in the fight against food waste. However, the small significance of GDP per capita and carbon footprint in model results shows the possible indirect influences or context-specific variables that might not be holistically captured by the current framework. These factors are open to further research in order to gain an in-depth understanding of the causes of food waste and to continue broadening the scope of the model.

The regression model of waste showed that household waste is the cause of total food waste per capita and therefore needs to be treated with priority as a field of intervention. In view of the result, effective recommendations can be drawn on how to handle food waste.

Policymakers should invest in initiatives that would reduce food waste at the household level. Public awareness through campaigns can educate consumers about the economic and environmental costs of food waste, besides promoting behaviors like meal planning, correct storage of food, and creative use of leftovers. Guidance on portion control and interpretation of best-before dates can help reduce the disposal of edible food. House-to-house composting incentives will be extended and supported to divert the organic waste to landfills for the same sustainability reasons.

It is here where retailers and manufacturers could most influence household food waste by offering better packaging and portion options. Smaller package sizes, resealable packaging, and clear labeling with storage and use tips could all serve to better manage the use of food. Supermarkets could also widen the availability of items close to the expiration date at a discount, in the hope that these are used, not wasted.

These can be further reinforced through community-level interventions; households can be allowed to give surplus food to the needy; community’s local governments and organizations are supposed to support increased access to programs for food redistribution; the organic material resultant of composting will go into agriculture/gardening, therefore to the waste diverted.

Beyond the household level, the lack of a robust relationship between total food waste and variables such as GDP per capita and carbon footprint also suggests that food waste reduction is independent of economic status or environmental impact. Therefore, policymakers should focus on tailored, practical solutions rather than assuming that general economic development or environmental policies will naturally address food waste. What is required is a collaboration between households, businesses, and local governments to provide a joined-up approach to the challenges presented by food waste.

### 5.3. Grouping Using the K-Means

**Figure 4:**
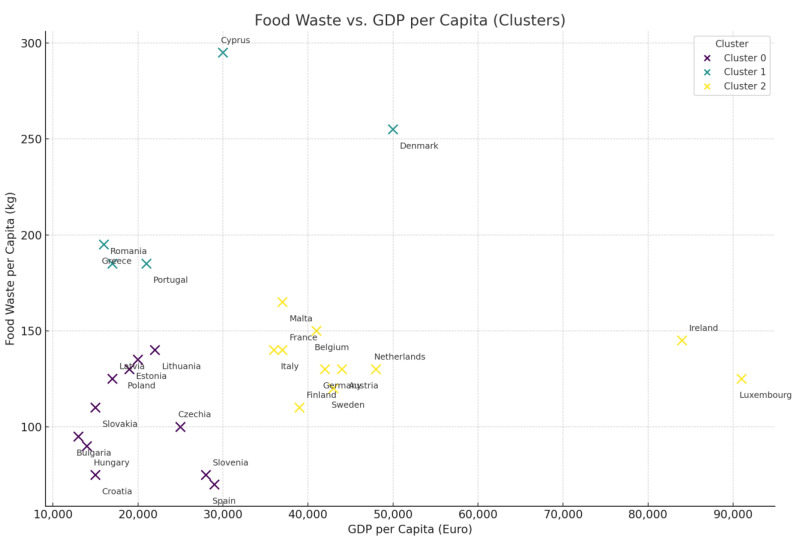
Cluster map grouping EU countries based on food waste patterns.

**Figure 5:**
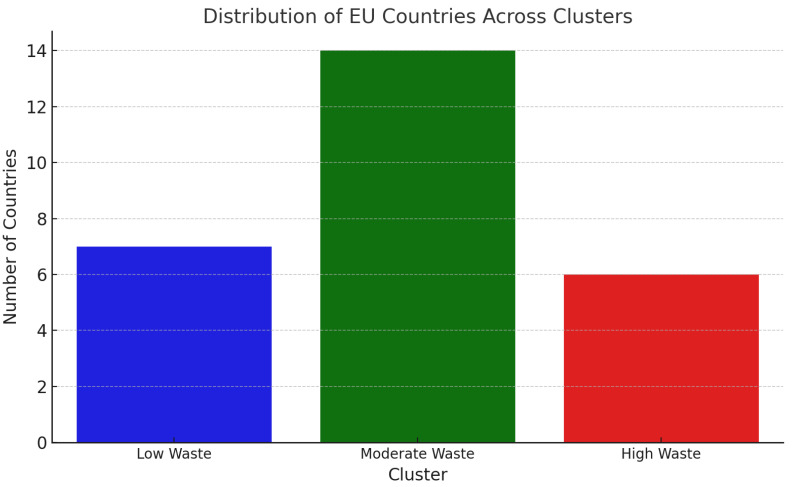
Distribution of EU countries across clusters.

With the help of cluster analysis, countries of the European Union have been grouped into evidence of patterns about food waste, with various sectors represented in standardized 2023 data and subjected to a k-means algorithm (Figure 4). Such contributions included primary production, manufacturing, retail distribution, restaurants, and households. Therefore, this has shown three kinds of clusters that the countries have because they have similar features on account of food waste. Examples are given in Table 9 and Figure 5.

The k-means cluster test clusters 0, 1, and 2 describe the different patterns of food wastage in EU countries by clustering countries on the basis of total per capita food wastage and industrial contribution. The low-wastage countries are represented by cluster 0 and have lower total per capita food wastage compared with the EU average. These countries, such as Spain, Slovenia, and Croatia, possess efficient food management systems, minimal domestic waste, and sound distribution channels that limit food loss along the supply chain to a minimum. Cluster 1 also comprises medium-waste countries such as Germany, France, and Belgium, where food wastage in all sectors is present but not excessive. They have well-established economies and structured food wastage policy regimes but are left with inefficiencies that are prevalent primarily at retail and household levels. Cluster 2 is made up of high-waste countries, namely Cyprus, Denmark, and Romania, and each of the countries has over 250 kg per capita food wastage attributed to high rates of loss during the production, retail, and final consumer levels influenced by inefficient supply chains, insufficient good waste management policy, and high rates of disposal among consumers.

Cluster 0 is low-waste countries in Figure 5. It is a per capita food wastage level cluster, i.e., Cluster 0 always produces the lowest cumulative amounts of waste, namely for the household and manufacturing sectors. These countries likely possess more effective consumer education programs, more effective portioning habits, and food donation and redistribution policies. Simultaneously, the countries in Cluster 2 are confronted with systemic inefficiencies that must be ironed out at different points along the food supply chain under duress. The outcome of clustering confirms that food wastage is not uniformly distributed across the EU but occurs in line with well-defined regional patterns, a conclusion that is supportive of policy intervention aimed at each cluster’s specific nature rather than a one-size-fits-all EU-wide approach.

First, there is a cluster of countries with a moderated level of total food waste, with predominance in the household sector. It includes Belgium, Germany, and France. These are quite well-balanced regarding sector contributions but high at the household level. This is typical for countries where food waste reaches average medium quantities: the situation is rather stable both from the point of view of waste management as well as the economic structure in general.

The second cluster is that of countries with high food waste: Denmark, Cyprus, and Romania. In real life, contributions are from more than one sector, like primary production and manufacture, in addition to households. For example, it is observed that Cyprus and Denmark have sharp manufacturing and retail inefficiencies that drive their positions within this high-waste cluster. This is systemic inefficiency or wider waste management challenges across the food supply chain.

The third cluster involves those countries that have overall low food waste: Spain, Croatia, and Finland. These countries present low contributions in almost all sectors, but most especially in sectors such as primary production and retail. Household waste remains a major contributor but is relatively low as compared with the other clusters. These are countries that have seemingly good practices in the management of waste and probably cultural or policy-driven behaviors that reduce all-over food waste.

These differences among clusters underline the complexity of the food waste problem in the EU: while most of the structural and high-level investments are needed in high-waste countries, more emphasis can be given to consumer behavior and household-level prevention of waste in moderate-waste countries. Low-waste countries represent examples of best practices in showing that quite substantial reductions in food waste are indeed achievable by concerted effort.

From the clustering analysis, customized interventions concerning the particular challenges of each group of countries come to light. For example, such cross-sector collaboration can be instrumental in high-waste countries to invest in technology that enhances efficiency, while for moderate-waste nations, interventions at the household level and improvement in food management practices can be undertaken. Results further cement the fact that reduction in food waste is not a one-size-fits-all affair and requires strategies that take into consideration regional and sectoral differences if any meaningful progress is to be made.

In the case of the moderate-waste cluster—for instance, for Belgium, Germany, and France—household waste is the dominant contributor, and intervention would more directly relate to consumer behavior and education. The policies that can be implemented include publicity campaigns on the environmental and economic impact of food waste, incentives for composting, education on portion control, proper food storage, and planning. Secondly, collaborating with retailers by selling portions and near-expiration items at lower prices may reduce food waste. Furthermore, enhancing the infrastructure for donating food will distribute the surplus to needy people.

In contrast, high-waste countries such as Denmark, Cyprus, and Romania require comprehensive and systemic intervention throughout the food supply chain, with particular emphasis on enhancing efficiency in primary production, processing, and retail. Investments in technology, such as precision agriculture and better inventory management systems, could reduce waste at the production and distribution stages. These inefficiencies can be improved through better oversight of production and storage practices; punitive measures against wasted food deemed avoidable might just spur businesses into sustainability actions. The partnerships with food banks or other food redistribution networks allow the excess food to be diverted away from landfills. This could further be complemented by deploying circular economic initiatives, which would find alternative industrial uses for by-products from the foods being created.

The agenda for countries in the low-waste cluster, such as Spain, Croatia, and Finland, should go more toward sustaining and further improving their current best practices. These countries can provide examples for the rest through successful strategies like community-based composting programs, sound regulatory frameworks, and public–private partnerships centered on sustainability. Further investment in education and innovative approaches has the potential to further reduce waste in this country. Such lofty ambitions can have impacts on neighbors simply due to the institution of similar measures.

There is an urgent need to strengthen the mechanisms of data collection and monitoring related to food loss at every step of the supply chain across clusters. This would, in turn, help policy thinkers to precisely identify points that require intervention and measure the performance of strategies laid down or implemented. Furthermore, regional cooperation in knowledge sharing between countries belonging to different clusters can accelerate progress while ensuring that effective initiatives are replicated across the board. Targeting specific interventions to the challenges and opportunities in each cluster, it is possible for the EU to take an important step toward reducing food waste and attaining its sustainability targets.

Low-waste countries, such as Spain and Croatia, have good food management systems, cultural practices that promote resourcefulness, and well-established policies that reduce waste. The countries have well-organized government programs, consumer education campaigns, and efficient redistribution networks that facilitate the diversion of surplus food to the vulnerable population. The country also has infrastructure that promotes composting and food donation, as well as societal practices that discourage wasteful consumption, which keeps food waste at its lowest.

On the other hand, Denmark and Cyprus waste somewhere in their supply chain of basic production from the point to consumption pattern and retail. The gigantic amount would be wasted through overproduction in the manufacturing stage, handling of stocks, bulk buying, and food quality mere cosmetic in consumption patterns. The reasons are the overbuying promotion marketing strategy and incorrect channel of redistribution. System inefficiencies and policy gaps within the countries involved ensure that the wastage of food is now no longer a matter of preference but a matter of structure that calls for an intervention that will transcend sectors.

Impacts of cluster analysis identify that interventions must be nation-focused as well as aim to address peculiar causes of wastage of food within each nation. Low-waste nations provide blueprints for activating consumer engagement and policy creativity, yet high-waste nations need to reorganize patterns of food production, processing, and eating. A one-size-fits-all policy to halt food waste will not work because country differences need special answers that resonate through the country’s economy, culture, and infrastructure environment.

### 5.4. Fixed Effects Model

The Fixed Effects Model (FEM) was employed to estimate the determinants of total food waste in European Union countries, controlling for country-specific characteristics that are time-invariant. Country-specific fixed effects are included in this model to eliminate the impact of unobserved heterogeneity so that the estimates capture the within-country variation in food waste.

The significant findings of the FEM (Fixed Effects Model) output are the perfect fit of the model (R^2^ = 1.000), and it suggests that the model explains all variations in total food waste. All coefficients of independent variables, such as GDP per capita, carbon footprint, food waste by households, food waste during manufacturing, and food waste during retail, are all similar to those in the multiple regression model, with all parameters identical.

The FEM (Fixed Effects Model) estimations indicate that some countries all the time display higher food wastage levels above what is predicted, even controlling for GDP per capita, carbon footprint, and industry-related generation of waste. Greece and Romania possess extremely high fixed effects, meaning that total food wastage per capita remains high even when controlling for other determinants. This suggests that either there are structural inefficiencies in their food distribution systems, insufficient enforcement of food waste prevention policies, or cultural consumption patterns leading to greater waste. Food waste in Romanian households is very high, as also indicated by the FEM (Fixed Effects Model) results suggesting that this country discards more food than would be explained by industrial and economic explanations alone.

Conversely, Hungary, Italy, and Portugal have negative fixed effects, which means that these countries generate less food waste per capita than anticipated by the independent variables of the model. This could represent more effective national waste reduction strategies, more knowledgeable consumers, or more effective food supply chains. Portugal, while having a relatively high household food waste rate, has lower overall fixed effects, suggesting maybe other sectors, manufacturing and retailing included, have improved their measures to reduce waste.

Denmark and Cyprus, two of the highest food-wasting countries in the sample, neither of them with extreme fixed effects. This shows that their excessive amounts of waste are largely explained by the independent variables—sectoral food waste contributions, GDP per capita, and carbon footprint—rather than by omitted country-specific heterogeneity. This is in contrast to Greece and Romania, where there is a great deal of unexplained heterogeneity. In Cyprus, food manufacturing and retail waste are the key contributors to food waste levels, whereas, in Denmark, the food industry is the key contributor to the total waste, as would be anticipated from their high coefficients in the multiple regression model.

Spain and Croatia, the EU’s lowest two rates of per-capita food waste, also tend to have predominantly neutral fixed effects. This is a hint that their lower-than-average food wastage can properly be explained through independent variables such as relatively lower waste in food terms at the manufacturing and retail levels. Their oiled machines of food chains, eating patterns, and plans for reducing food wastage all appear to fit anticipated wastage levels and account for very little leftover unexplained variance.

France and Germany, being two of the largest economies within the EU, have near-zero fixed effects that indicate their generation of food waste is as expected based on their GDP, carbon footprint, and industry-specific wastage. This suggests that policies in these countries, while successful in keeping the waste at predicted levels, do not stray from the overall EU trends.

### 5.5. Discussion of Results with Literature

Results of this research indicate the presence of stark regional disparities in food wastage generation among the member states of the EU, with some states boasting functioning waste management systems and other member states experiencing dysfunctional food supply systems. Such levels of per capita food wastage in Cyprus and Denmark, and to a certain extent the final outcomes of inefficiencies in retail and manufacturing, also correlate with existing literature identifying excessive production, logistic failure, and high standards of aesthetic food quality as determinants of excessive loss among industrialized countries [61,63]. Conversely, Spain and Croatia boast some of the EU’s lowest food wastage rates, which is aligned with evidence that indicates that high-performing national policies, public education campaigns, and efficient redistributive chains can be held responsible for preventing wastage of food [62,66]. This lends proof to the premise that policy measures and public consciousness campaigns can potentially prevent food waste in retailers as well as at the household level.

Another important finding of this study is that food wastage in domestic settings is the highest contributor amongst all the EU countries, regardless of whether economic differences between the nations are being considered or not. This is evidenced by Thyberg and Tonjes [66], who concluded that the household was generating more than 50% of food wastage in the majority of the high-income nations because of over-purchasing, confusion of sell-by dates, and poor meal planning. The research also implies that GDP per capita has little effect on overall food waste, as assumed by Caldeira et al. (2019) [65], who stated that economic prosperity is not necessarily coupled with more or less waste but policy and consumer culture are more fundamental. This requires consumer-targeted interventions, such as routine expiry date labeling and food portioning and storage health promotion programs.

From the perspective of supply chain management, data supporting over-abundance waste within Danish and Cypriot retail and manufacturing industries are in tandem with information stating that processing inefficiency and overproduction as stockpile inventory are primary factors within wastage in food at country levels [60,61]. This informs the case for food firms to adopt dynamic pricing, improve forecasting, and maximize redistribution schemes in a bid to minimize waste. This essay’s use of clustering analysis to group EU countries by food waste behavior brings valuable depth to the case that patterns of waste generation differ with economic and regulatory contexts and supports regionally tailored solutions rather than one-size-fits-all [62].

In the circular economy and sustainability context, the research contributes to the discussion of how reducing food waste is weighed against environmental policy measures such as the European Green Deal and the UN SDGs. In reliance on De Laurentiis’s [66] study, the current work grounds its supposition that France and Spain experience less food loss after the fact that they have strong policy interventions while their weaker counterparts with weak enforcement lag behind. This substantiates the argument that waste minimization activities need to be supported by master policies on sustainability, i.e., food donation incentives, tax credits for food recovery ventures, and more stringent regulation of waste reporting.

The theoretical model that can be used to account for consumer-related determinants of food wastage is the Theory of Planned Behavior (TPB) [79,80], which can be used to account for how the behavior of a specific individual is explained by three salient determinants: attitudes, subjective norms, and perceived behavioral control. Conceptualizing TPB in the context of the research findings in this study gives a better picture of the determinants of behavior toward food waste and implies intervention channels.

The TPB proposes that behavior attitudes have an important influence on intention and behavior. The research indicates that wastage of food is mainly caused by over-purchasing, poor food storage management, and ignorance regarding the meaning of expiration dates. These behaviors are a result of the attitude that there is sufficient food and that it is easily replaceable. Consumers in EU high-food-waste nations such as Cyprus and Denmark hold weak negative attitudes towards food waste. Spain and Croatia, for example, the low-food-waste nations, may instead, however, maintain culture- or policy-driven practices of averagely greater consumption. Interventions are required to cover remolding attitudes by unveiling consumers to the environmental- and money-linked costs of food waste, bolstered by publicity campaigns and educational strategies.

Subjective norms refer to perceived social pressure to do or not to do a particular behavior. Social norms play a role in food wastage in some EU (European Union) member states, as found in the research. For example, cultural norms of food surpluses with respect to food and looks standards of food with respect to food choice can affect high-waste nations’ food consumption habits. On the other hand, countries with well-developed food redistribution programs or strong social movements for sustainability have lower levels of food loss. Encouraging social norms to use food rationally—e.g., food-sharing activities at the community level and government-backed donation programs—can discourage waste. Suppliers and retailers can also influence norms by selling “ugly” but safe food at lower prices to reshape food quality perceptions.

Perceived control of behavior is the perception that the person can carry out a specific behavior. Systemic inefficiencies in food distribution, labeling confusion, and inappropriate knowledge in storage areas are quoted by the study as the primary reasons for food wastage. Consumers in countries with high food waste may believe they are outside their control with regard to reducing food waste due to misleading “use by” dates or the absence of access to redistribution programs. Technology-driven remedies, meal planning programs, and more informative labeling can heighten consumers’ perceptions of control of waste minimization. Governments and companies can strengthen food management through simplification of storage guidance, facilitating easier donation of more foods, and encouraging the purchase of near-expiration products.

According to TPB (Theory of Planned Behavior), this calls for targeting consumer attitudes toward food waste. Some of the attitudes that contribute to the problem include viewing food waste as insignificant or the belief that discarded food cannot be recovered [68,69]. The key drivers in such cases must, therefore, be tackled through education campaigns focusing on environmental and economic costs due to food wastage, besides the moral imperative of reduction in waste. Linking food waste, for example, to greenhouse gas emissions or resource depletion can help change public perception and create more sustainable attitudes.

The paper identifies systemic inefficiencies and logistical challenges as key contributors to food waste. These often diminish perceived behavioral control over food waste, especially in regions with poor infrastructure for waste management or limited access to redistribution networks. Perceived behavioral control reflects an individual’s belief in his or her ability to perform a behavior. Interventions that can help enable this include meal planning mobile phone applications, good storage of food, and extended policies to redistribute surplus food or compost waste, thereby enabling better choices by consumers. For instance, clearer labeling in order to overcome confusion between the “use by” and “best before” dates directly addresses one of the deficits in control related to food disposal practices.

The TPB framework emphasizes interrelations between attitudes, subjective norms, and perceived behavioral control; for instance, reducing household waste requires changing individual attitudes and developing social norms against wastefulness, along with enhancing systems that facilitate good food management [81,82,83]. In this study, the clustering analysis showed that countries with high waste, like Cyprus and Denmark, need systemic interventions, whereas low-waste countries, like Spain, are exemplary in how well societal and structural alignment reduces waste.

This research evidence has shown that while all other socio-economic and environmental factors are important, individual and collective behavior backed by the TPB framework holds the key to reducing food waste in the European Union by a high margin.

## 6. Conclusions

### 6.1. Research Questions Response

The most significant sectoral drivers of food loss for the European Union from 2021 to 2023 were marked by domestically caused waste, then from the manufacturing industry, retail industry, and food service industry (Q1). In the total of all EU (European Union) countries combined, household food wastage always made up the larger share, ranging from some 55 kg/capita in low-waste countries like Croatia to over 100 kg/capita in high-waste nations like Italy and Portugal. This is the basis of the central position of consumption habits in food wastage via such mechanisms as overbuying, storage, and misreading use-by dates. Manufacturing and reselling food waste, nevertheless, varied widely across nations. Manufacturing waste was very high in Denmark and Cyprus due to inefficiency in food processing and supply chain management, earning them the top spot as among the highest wastage countries in the EU. Retail waste, while overall lower in volume than household and production waste, was nonetheless a problem, particularly where there were poor inventory control and overproduction practices.

Food waste patterns differed across regions, with some nations having more waste reduction practices than others. Spain and Croatia consistently recorded the lowest per capita food waste, reflecting successful policy interventions and consumer education initiatives that guaranteed reducing household and retail waste. The highest food waste was recorded by Cyprus, Denmark, and Romania, largely because of inefficiencies across different sectors such as primary production, manufacturing, and retail. These differences suggest that although waste minimization at the household level is a problem to be tackled for all member nations, sector-specific interventions are necessary to tackle nation-specific inefficiencies. Better food production, logistics of distribution, and retail control will reduce waste most effectively in the highly wasted nations, while consumer education and more stringent retail controls would best fit those nations with intermediate levels of waste like Germany and France. That such levels of domestic waste are still high throughout the EU is a sign that there needs to be common policy action and changed behavior instated in an effort to stop food waste at its most crucial origin.

For 2021–2023, the highest per capita food wastage in the European Union was in Cyprus, Denmark, and Romania (Q2). The highest per capita food wastage occurred in Cyprus, with the highest levels of 294 kg in 2023, primarily owing to primary production (52 kg), food manufacture (72 kg), and retail loss (60 kg). Food processing inefficiencies, stockholding problems, and ineffective retailing practices were the main causes of this undue waste. Similarly, Denmark saw an increase in total food waste from 221 kg per capita in 2021 to 254 kg per capita in 2023, mainly because of overproduction waste in the manufacturing sector, which accounted for 118 kg per capita in 2023. Overproduction in Romania was due to inefficiencies in primary food production and household waste, totaling 99 kg per capita in 2023. The common causes of such high-waste countries were overproduction, inefficient supply chain management, and inefficient waste reduction policies across sectors.

Meanwhile, Spain and Croatia still had the lowest per capita food losses, where Spain reduced the level from 69 kg per capita in 2021 to 65 kg per capita in 2023, while Croatia had a lower rate consistently of 72 kg per capita for 2021 and 2023. Household waste was much lower for other EU countries in Spain and Croatia and amounted to some 26 kg per capita in Spain and 55 kg per capita in Croatia in 2023. These reduced levels of food waste might have been due to more successful consumer education campaigns, better household food management, and effective food redistribution programs. In addition, improved retail and distribution systems in the countries mentioned above enabled waste reduction at the manufacturing level and in retail outlets. These trends suggest that while food waste by consumers is still the dominant trend in all the EU member states, differences in policy response, infrastructure, and consumer mindset play a significant role in determining the level of food wastage.

There are also regional variations in food waste trends among EU countries, with Southern and Eastern European countries having lower levels of waste than Northern and Western European countries with significantly higher per capita food waste (Q3). Spain and Croatia, for instance, had the lowest waste rates consistently, around 65–72 kg per capita, due to good household food management, consumer education, and efficient redistribution channels. On the other hand, the highest amounts of waste were observed in Cyprus and Denmark, more than 250 kg per capita, as a result of extensive losses in retail, industry, and first production. Germany and France, falling in the intermediate category, experienced historical food waste problems, with the majority happening at the household level, indicating stronger consumer behavioral interventions. The differences occur because of differences in efficiency in the food sector, supply chain functions, eating patterns, and policy efficiency in a nation to avoid food wastage.

Policy measures for food wastage at different stages of the supply chain need to be region-specific and sector-specific. For countries with high wastage rates like Denmark and Cyprus, policy interventions need to be directed towards raising the productivity of retailing and production, e.g., better forecasting arrangements, stricter controls on stockpiling, and incentives for surplus food donations. But in middle-waste countries like France and Germany, home interventions would take precedence, including advertising campaigns, rewarding food-sharing establishments, and enhanced date marking. Low-waste countries like Spain and Croatia must meanwhile continue to uphold robust waste reduction efforts and demonstrate the way forward as best practice role models in the EU. There is a need for a coordinated EU-wide policy, with laws, economic incentives, and public awareness, to harmonize action and ensure sustainable food management throughout all member states.

The analysis confirms significant regional disparities in food waste levels across EU countries, driven by economic development, consumer behavior, and policy implementation (Hypothesis H1). Countries such as Spain and Croatia consistently report lower food waste per capita (around 65–72 kg), largely due to effective household management, consumer awareness programs, and well-structured food redistribution policies. In contrast, high-waste nations like Cyprus and Denmark (with over 250 kg per capita) experience substantial losses in manufacturing, retail, and primary production, indicating inefficiencies in supply chain management and a lack of stringent waste reduction policies. Additionally, GDP per capita does not strongly correlate with food waste levels, as high-income countries like Germany and France generate waste at similar levels to lower-income nations like Romania. Instead, consumer habits, waste management infrastructure, and national policies play a more decisive role. The findings suggest that countries with stronger regulations, such as food waste reporting mandates and donation incentives, tend to manage food waste more effectively, whereas those with weak enforcement or systemic inefficiencies experience higher losses across the supply chain.

### 6.2. Theoretical Implications

The theoretical contributions in this article contribute to the accumulating stock of research on food waste, applying behavioral economics, environmental sustainability theory, and supply chain management frameworks to explain patterns in European Union food waste. Findings validate the TPB that consumer attitude, social norms, and perceived personal control over behavior are significant determinants of household food waste, and interventions based on these psychological determinants decrease waste. The study validates systems theory as well in the sense that food waste is not a problem based on linearity, but rather, it is a problem of systems, which requires multi-level concerted action at production, distribution, and consumption stages. Integrating the application of regression modeling and cluster analysis, this study also breaks new ground in quantitative research on food wastage studies and provides a quantifiable platform to conduct future research on the cross-link between economic indicators, environmental impacts, and industry-specific trends of wastage. These results provide a starting point for researchers and policymakers to design more integrated, empirical steps toward mitigating food wastage at national and EU levels.

### 6.3. Practical Implications

The research policy implications acknowledge the need for targeted food waste mitigation measures at different levels of the supply chain, with a particular focus on household, retail, and manufacturing operations. The policymakers can apply these findings in the development of region-specific policies, such as public campaigns, better-enforced food labeling regulations, and food donation incentives to mitigate household waste. For Denmark and Cyprus, which are both part of high-wastage countries, streamlining supply chain coordination, streamlining stock management, and establishing circular economy principles can all minimize food wastage in manufacturing and retailing operations by notable proportions. Food businesses, especially businesses, can utilize traceability development and waste redistribution technologies, whereas local consumers can plan meals more efficiently and store food. By harmonizing national policies with the EU sustainability goals, the research offers a gateway to adopting better waste management regimes that will bring economic gains, reduced environmental footprints, and enhanced food security in the European Union.

### 6.4. Limitations

Its main limitations really emanate from the scope and nature of data used, methodologies, and analytical frameworks. Even while the given study provides quite an enlightening overview of the patterns of food waste across the European Union, it tends to rely on aggregated data that perhaps do not represent the full local nuances in the management of waste, cultural behaviors, and implementations of policies for each country in particular. The granularity at which this is done is essential to the understanding of microlevel driving factors of food losses within particular contexts. Another limitation is dependence on socio-economic indicators, such as GDP per capita and carbon footprint, to explore drivers of food waste.

## Figures and Tables

**Table 1:** Environmental and economic challenges associated with food waste.

Aspect	Challenges	Examples/Impacts
Greenhouse gas emissions	The decomposition of food in landfills produces methane, a powerful greenhouse gas.	Food waste is estimated to be one of the biggest causes of climate change, at around 8–10% globally of estimated greenhouse gas emissions.
Resource depletion	Wasted food represents the waste of water, energy, and land that are used in the production and distribution cycle.	Agriculture uses up approximately 70% of global freshwater; a lot of this is for food that is wasted.
Ecosystem degradation	Overproduction and waste spur deforestation, soil erosion, and loss of biodiversity.	Forests are being cut down for agriculture; this leads to habitat loss and the loss of species.
Pollution	Food wastage leads to water and soil pollution, in addition to contributing to inefficiency in waste management operations.	Nutrient runoff from wasted food can cause algal blooms and water contamination, affecting aquatic ecosystems.
Household costs	Over-purchasing and incorrect storage of food items cause financial losses to households.	The average household wastes about EUR 600 in food in a year.
Business losses	Unsold inventory and overproduction result in costs in retail and food service sectors.	Restaurants waste vast quantities of prepared but unconsumed food at huge losses
National economic impact	Food waste denies national food security and leads to increased public spending on the management and disposal of waste.	Governments spend colossal amounts on managing landfill waste from food that could be diverted to other, more meaningful areas.
Missed opportunities	Food waste could instead be used to feed populations or repurposed for industries like bioenergy.	It is estimated that the Commission loses around 88 million tons every year in waste, which accounts for an approximate loss of EUR 143 billion annually in the EU alone.

Source: Author’s own work on the basis of [3,4,5,6,7,8,9,10,11,12,13,14,15,16,17,18,19,20,21,22,23,24,25,26,27,28,29,30,31].

**Table 2:** Summary of data sources and methods used in this study.

Item	Period	Unit	Data Source	Brief Description
Total Food Waste per Capita	2021–2023	kg per capita	Eurostat, Statista	Annual data on total food waste per capita collected from national statistical reports.
Household Food Waste	2021–2023	kg per capita	Eurostat, Statista	Estimated based on self-reported surveys and national food consumption studies.
Manufacturing Food Waste	2021–2023	kg per capita	Eurostat, Statista	Derived from industry reports, food processing sector statistics, and waste audits.
Retail Food Waste	2021–2023	kg per capita	Eurostat, Statista	Based on supermarket inventory records, disposal logs, and food waste declarations.
Primary Production Waste	2021–2023	kg per capita	Eurostat, Statista	Includes waste generated in agriculture, fishing, and primary food production industries.
Carbon Footprint	2021–2023	Tons of CO_2_ equivalents per capita	Eurostat, Statista	Estimates calculated using national energy use statistics and emission factors.
GDP (Gross Domestic Product) per Capita	2021–2023	Euros per capita	Eurostat, Statista	GDP per capita data extracted from national economic databases and adjusted for inflation.
Clustering Analysis	2021–2023	Categorical	Eurostat, Statista	K-means clustering applied to categorize countries based on food waste patterns across sectors.
Regression Analysis	2021–2023	Statistical Coefficients	Eurostat, Statista	Multiple linear regression analysis performed to assess the impact of economic and environmental factors on food waste.

**Table 3:** Food waste by sectors in 2021 [kilograms per capita].

	Total (Aggregate Changing According to the Context)	Primary Production of Food—Agriculture, Fishing and Aquaculture	Manufacture of Food Products and Beverages	Retail and Other Distribution of Food	Restaurants and Food Services	Total Activities by Households
European Union—27 countries (from 2020)	128	13	22	9	13	71
Spain	69	18	11	6	4	30
Croatia	73	10	3	1	4	55
Slovenia	74	0	5	7	24	37
Czechia	91	3	9	6	4	69
Hungary	94	2	19	4	2	66
Slovakia	107	13	23	5	1	65
Finland	113	6	29	10	14	53
Bulgaria	114	10	20	7	19	58
Poland	120	18	21	14	7	61
Sweden	121	10	29	11	9	61
Estonia	125	18	24	15	8	61
France	129	18	26	10	16	60
Germany	131	2	19	9	23	78
Italy	136	12	9	6	3	107
Austria	136	2	19	9	23	83
Lithuania	137	29	10	10	2	86
Latvia	145	17	19	8	19	82
Belgium	146	3	54	11	8	70
Luxembourg	147	12	17	14	14	91
Ireland	152	11	44	14	35	48
Malta	160	1	15	8	45	92
The Netherlands	161	27	59	12	5	59
Romania	166	36	16	2	25	86
Portugal	175	10	6	21	16	123
Greece	191	35	35	14	21	87
Denmark	221	11	102	17	11	79
Cyprus	273	49	67	56	30	71

Source: on the basis of data from [67].

**Table 4:** Food waste by sectors in 2022 [kilograms per capita].

	Total (Aggregate Changing According to the Context)	Primary Production of Food—Agriculture, Fishing, and Aquaculture	Manufacture of Food Products and Beverages	Retail and Other Distribution of Food	Restaurants and Food Services	Total Activities by Households
European Union—27 countries (from 2020)	129	13	22	9	13	71
Spain	67	18	11	6	5	28
Croatia	72	10	2	1	4	55
Slovenia	73	0	6	7	22	38
Hungary	92	1	17	6	2	66
Slovakia	101	4	23	5	2	66
Czechia	108	3	18	7	15	64
Bulgaria	109	9	20	6	18	56
Sweden	118	10	29	10	9	59
Poland	122	17	22	14	7	62
Finland	122	5	29	10	15	63
Luxembourg	127	11	18	14	14	69
Estonia	128	14	31	10	9	64
France	129	18	25	9	16	60
Latvia	130	16	17	9	15	73
Germany	131	2	18	9	22	79
Austria	134	1	21	9	20	83
Lithuania	139	29	10	12	2	86
Italy	140	11	9	8	4	107
Ireland	147	10	42	15	37	43
The Netherlands	148	18	65	12	5	48
Belgium	149	3	58	10	8	70
Malta	156	1	15	7	45	88
Romania	177	37	14	2	31	94
Portugal	181	13	7	22	16	124
Greece	193	34	38	14	20	86
Denmark	230	10	104	17	12	87
Cyprus	285	50	70	59	32	74

Source: on the basis of data from [67].

**Table 5:** Food waste by sectors in 2023 [kilograms per capita].

	Total (Aggregate Changing According to the Context)	Primary Production of Food—Agriculture, Fishing and Aquaculture	Manufacture of Food Products and Beverages	Retail and Other Distribution of Food	Restaurants and Food Services	Total Activities by Households
European Union—27 countries (from 2020)	127	12	23	10	14	68
Spain	65	16	11	7	6	26
Slovenia	71	0	5	7	26	33
Croatia	72	10	2	1	4	55
Hungary	84	1	14	6	2	60
Bulgaria	95	10	23	6	15	41
Czechia	101	1	15	6	17	61
Slovakia	106	7	26	6	2	65
Finland	109	5	25	10	15	55
Sweden	117	9	29	10	14	56
Luxembourg	122	12	18	13	15	65
Poland	123	20	15	13	7	69
Latvia	124	14	16	8	13	71
Germany	129	2	19	9	24	75
The Netherlands	129	18	50	9	5	48
Austria	131	1	23	9	28	70
Estonia	134	16	29	15	10	65
Lithuania	138	29	10	12	2	85
France	139	17	35	12	16	58
Italy	139	11	9	11	8	100
Ireland	144	10	44	17	30	42
Belgium	151	3	63	11	10	64
Malta	162	1	14	9	51	88
Romania	181	32	20	2	29	99
Portugal	184	11	6	22	23	123
Greece	194	34	9	14	21	86
Denmark	254	20	118	17	13	86
Cyprus	294	52	72	60	33	77

Source: on the basis of data from [67].

**Table 6:** GDP per capita and carbon footprint per country.

	GDP per Capita [Euro]	Carbon Foodprint [Tons of CO_2_ Equivalent (tCO_2_e) per Person]
European Union—27 countries (from 2020)	32,000	7.2
Belgium	44,460	8.0
Bulgaria	13,300	5.0
Czechia	26,110	11.0
Denmark	47,700	7.5
Germany	41,490	9.0
Estonia	19,250	12.1
Ireland	83,300	10.0
Greece	14,430	7.0
Spain	25,620	6.0
France	36,760	6.0
Croatia	15,250	5.0
Italy	33,750	7.0
Cyprus	29,080	7.0
Latvia	14,880	5.0
Lithuania	22,130	5.0
Luxembourg	90,900	14.5
Hungary	15,330	5.0
Malta	37,340	4.2
The Netherlands	51,500	8.0
Austria	46,520	8.0
Poland	18,480	9.0
Portugal	19,460	5.0
Romania	16,710	4.7
Slovenia	28,880	5.0
Slovakia	15,020	6.0
Finland	37,860	9.0
Sweden	44,620	6.0

Source: on the basis of data from [68,69].

**Table 7:** Regression model parameters.

Variable	Coefficient	Std. Error	t-Statistic	*p*-Value
Constant	13.5621	14.8434	0.914	0.371
GDP per capita	0.0000793	0.000210	0.377	0.710
Carbon footprint	−1.3580	1.5553	−0.873	0.392
Household food waste	1.1964	0.1544	7.751	0.000
Manufacturing food waste	0.9133	0.1507	6.059	0.000
Retail food waste	2.1079	0.3460	6.090	0.000

**Table 8:** Sensitivity analysis of the model.

Variable	Change (%)	Mean Impact on Total Food Waste
GDP per capita	5%	0.1306
Carbon footprint	5%	−0.4903
Household food waste	5%	4.0399
Manufacturing food waste	5%	1.2118
Retail food waste	5%	1.2497
GDP per capita	10%	0.2612
Carbon footprint	10%	−0.9807
Household food waste	10%	8.0797
Manufacturing food waste	10%	2.4235
Retail food waste	10%	2.4993

**Table 9:** The clustering results.

	Primary Production	Manufacturing	Retail Distribution	Restaurants	Household Waste	Total Food Waste	Cluster
Belgium	3	63	11	10	64	151	1
Bulgaria	10	23	6	15	41	95	1
Czechia	1	15	6	17	61	101	1
Denmark	20	118	17	13	86	254	2
Germany	2	19	9	24	75	129	1
Estonia	16	29	15	10	65	134	1
Ireland	10	44	17	30	42	144	1
Greece	34	9	14	21	86	194	2
Spain	16	11	7	6	26	65	0
France	17	35	12	16	58	139	1
Croatia	10	2	1	4	55	72	0
Italy	11	9	11	8	100	139	1
Cyprus	52	72	60	33	77	294	2
Latvia	14	16	8	13	71	124	1
Lithuania	29	10	12	2	85	138	1
Luxembourg	12	18	13	15	65	122	1
Hungary	1	14	6	2	60	84	0
Malta	1	14	9	51	88	162	2
The Netherlands	18	50	9	5	48	129	1
Austria	1	23	9	28	70	131	1
Poland	20	15	13	7	69	123	1
Portugal	11	6	22	23	123	184	2
Romania	32	20	2	29	99	181	2
Slovenia	0	5	7	26	33	71	0
Slovakia	7	26	6	2	65	106	0
Finland	5	25	10	15	55	109	0
Sweden	9	29	10	14	56	117	0

## Data Availability

The original contributions presented in this study are included in the article. Further inquiries can be directed to the corresponding author.
